#  Neurolymphomatosis of Brachial Plexus in Patients with Non-Hodgkin's Lymphoma

**DOI:** 10.1155/2013/492329

**Published:** 2013-11-12

**Authors:** Yong Jun Choi, Jung A. Shin, Yong Hoon Kim, Soon Joo Cha, Joong-Yang Cho, Seung Hee Kang, Seong Yoon Yi, Hye Ran Lee

**Affiliations:** ^1^Department of Internal Medicine, Inje University Ilsan Paik Hospital, 170 Juhwa-ro, Ilsanseo-gu, Goyang-si, Gyeonggi-do 411-706, Republic of Korea; ^2^Department of Radiology, Inje University Ilsan Paik Hospital, 170 Juhwa-ro, Ilsanseo-gu, Goyang-si, Gyeonggi-do 411-706, Republic of Korea; ^3^Department of Neurology, Inje University Ilsan Paik Hospital, 170 Juhwa-ro, Ilsanseo-gu, Goyang-si, Gyeonggi-do 411-706, Republic of Korea; ^4^Department of Radiation Oncology, Inje University Ilsan Paik Hospital, 170 Juhwa-ro, Ilsanseo-gu, Goyang-si, Gyeonggi-do 411-706, Republic of Korea

## Abstract

Neurolymphomatosis (NL) is a rare clinical disease where neoplastic cells invade the cranial nerves and peripheral nerve roots, plexus, or other nerves in patients with hematologic malignancy. Most NL cases are caused by B-cell non-Hodgkin's lymphoma (NHL). Diagnosis can be made by imaging with positron emission tomography (PET) and magnetic resonance imaging (MRI). We experienced two cases of NL involving the brachial plexus in patients with NHL. One patient, who had NHL with central nervous system (CNS) involvement, experienced complete remission after 8 cycles of R-CHOP (rituximab, cyclophosphamide, doxorubicin, vincristine, and prednisone) chemotherapy but relapsed into NL of the brachial plexus 5 months later. The other patient, who suffered from primary central nervous system lymphoma (PCNSL), had been undergoing chemoradiotherapy but progressed to NL of the brachial plexus.

## 1. Introduction

Neurolymphomatosis (NL) is the term for nerve infiltration by neurotropic neoplastic cells in non-Hodgkin's lymphoma (NHL) or acute leukemia [1]. Although the precise incidence is not known, it is estimated to occur in about 0.2% of all NHL patients [1]. NL can occur as a primary presentation of NHL but is more often seen when lymphoma disseminates into the peripheral nervous system from systemic sites or the CNS [2]. The presenting symptoms are diverse, depending on the sites involved. Diagnosis can be difficult since NL may mimic many conditions, and a clinical or histopathological diagnosis may not be established until biopsy. For the diagnosis, parallel use of magnetic resonance imaging (MRI) and positron emission tomography-computed tomography (PET-CT) can be effective, though biopsy of the involved structure is the gold standard method for diagnosis. PET-CT in particular appears to be a highly sensitive tool to diagnose NL [2, 3]. We report here on two cases of NL that developed in the brachial plexus, diagnosed by MRI and PET-CT.

## 2. The Case Report

**Case 1**

**Figure 1:**
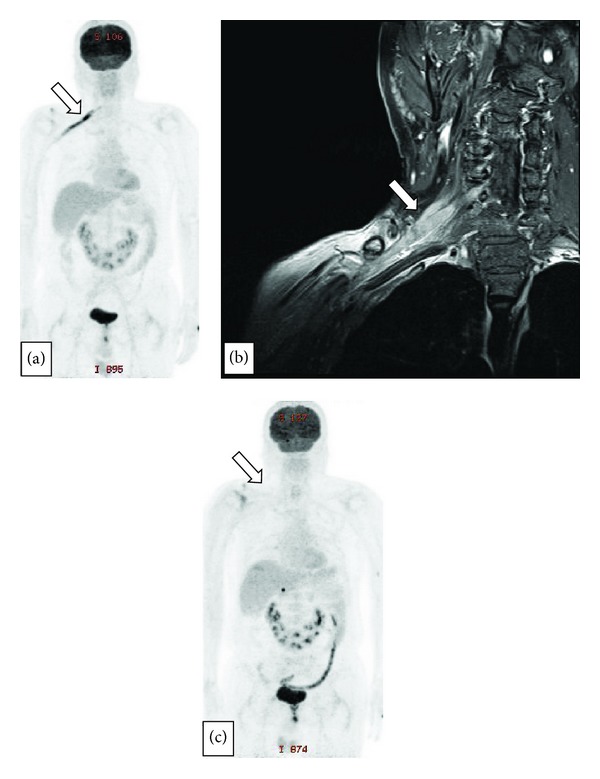
(a) PET-CT. Strong linear FDG uptake along the right brachial plexus was found, which had not been seen on PET-CT performed 2 months ago (white arrow). (b) Cervical spine MRI with contrast enhancement. The trunk of the right brachial plexus on the level of C5 and C6 were thickened with heterogeneous enhancement, suggesting lymphoma infiltration. (c) Follow-up PET-CT. Right brachial plexus invasion was improved after chemoradiotherapy, but new lesions were seen in right temporal base, right sciatic nerve, and right adrenal gland.

A 60-year-old male patient came to the hospital complaining of right shoulder and upper arm pain with weakness that had appeared a month before. He had been diagnosed with stage IV diffuse large B-cell lymphoma (DLBCL) of the prostate 6 months previously. At the time when he was diagnosed with lymphoma, it had spread to the whole body, involving cranial nerves III, V, and VI. After 8 cycles of systemic chemotherapy with a R-CHOP regimen and intrathecal chemotherapy, he achieved complete remission and was free from disease until the right shoulder pain developed. In neurologic examination, measurement by manual muscle test found the muscular strength of the flexor of the right shoulder, adductor, abductor, and elbow extensor to be poor. However, the reflexes of the two arms were normal, and there was no hypoesthesia in a sensory test. No abnormal findings were revealed from a complete blood count, biochemical blood test, and blood coagulation test. However, polyneuropathy involving musculocutaneous nerve, axillary nerve, median nerve, and ulnar nerve in the branch level of the right brachial plexus was suspected from the nerve conduction velocity test. Accordingly, an MRI scan of the right shoulder was performed, but there was no evidence of neuropathy. The pain he experienced was considered to be postherpetic neuropathy because he had suffered from herpes zoster on C5 dermatome 5 months ago. Therefore conservative care and rehabilitation were undertaken. In spite of conservative management, his motor weakness in elbow flexor and wrist extensor got worse from grade 3 to grade 1. And hypoesthesia appeared in C4~T1 dermatome, especially marked in C5~C6 level. A cerebrospinal fluid (CSF) study and brain MRI were done to identify progression of disease. The CSF examination showed neither signs suggesting infection nor malignant tumor infiltration. No actual brain lesion was found, and the previous remission status seemed to have been maintained, according to the brain MRI. But PET-CT revealed strong FDG (fluorodeoxyglucose) uptake along the right brachial plexus (Figure 1(a)). The venous Doppler sonography was carried out to identify thrombosis in the right subclavian vein, but none was observed. Under the suspicion of neuroinvasion of lymphoma, cervical MRI was conducted, which showed the thickening and heterogeneous enhancement of C5~C6 nerves, the brachial plexus trunk (Figure 1(b)). Therefore, we were able to diagnose neurolymphomatosis. Following diagnosis, he was treated with intrathecal chemotherapy with methotrexate, hydrocortisol, and cytarabine, followed by radiotherapy on right brachial plexus with a total dose of 3600 cGy divided to 18 fractions and rehabilitation. His arm weakness recovered almost completely after treatment. Then, systemic chemotherapy with a regimen containing a high dose of methotrexate was administered. The right brachial plexus invasion was improved, but multiple new lesions involving the right temporal base, right sciatic nerve, and right adrenal gland developed, as shown in follow-up PET-CT (Figure 1(c)). Despite aggressive chemotherapy, his disease progressed extensively, and he died 5 months after his initial diagnosis of NL.

**Case 2**

A 50-year-old female patient was diagnosed with primary CNS lymphoma (PCNSL) involving the left basal ganglion. PCNSL was pathologically confirmed as DLBCL. Her disease achieved partial remission after 10 weeks of scheduled chemotherapy with methotrexate/procarbazine/vincristine, followed by radiotherapy. While undergoing a 5-week course of whole brain radiotherapy, she began to complain of right arm weakness and pain, which gradually worsened. There was no trauma history that could be related to her symptoms. Neurologic examination of the right arm showed slightly compromised fine motor and sensory capabilities. Manual muscle testing (MMT) of right upper arm muscles revealed grade 4~5. Brain MRI was performed to identify seeding, infarction, or the regrowth of brain lesions. The MRI showed that the mass size of the previous lesion in the left basal ganglion was much reduced, and a CSF study did not identify CNS lymphoma progression or infection.

We decided to observe the patient because her arm pain was thought to have developed from vigorous rehabilitation, and it was controlled by analgesics. However, the weakness and hypoesthesia of her arm developed further.

**Figure 2:**
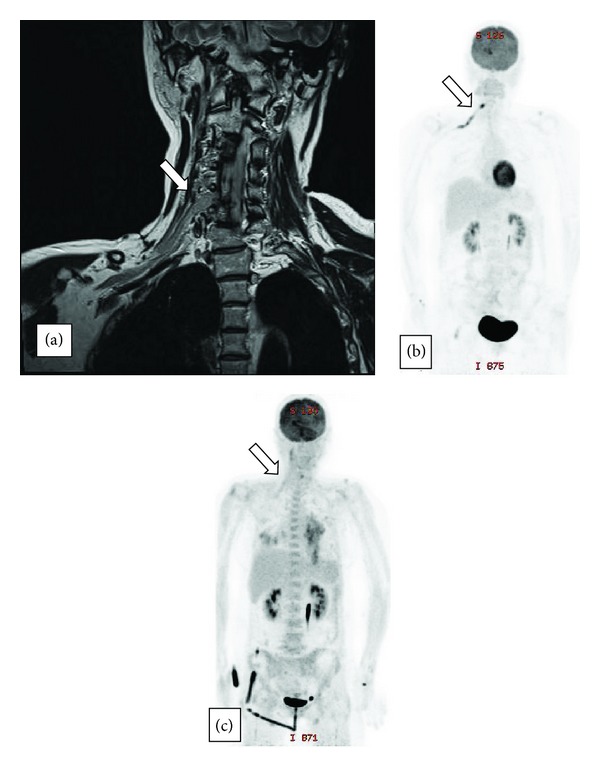
(a) Cervical spine MRI (T2-weighted image). Cervical spine MRI demonstrated a 0.7 × 0.9 cm sized mass (white arrows) in the extraforaminal portion of C5 and C6, with T2 high signal intensity. Nerve roots of C5 and C6 were compressed by the mass, but there was no involvement of the spinal canal or epidural space and bone. These features were not found on the cervical MRI performed 5 months ago. (b) PET-CT. Strong linear FDG uptake along the right brachial plexus and strong focal FDG uptake in the extraforaminal portion of C5 and C6 was seen. (c) Follow-up PET-CT. There was no abnormal FDG uptake in the previous right brachial lesion after chemoradiotherapy.

Under the follow-up neurologic examination, decreased exercise ability was found to have progressed, especially in the C5 region from grade 4 to grade 2, and severe hypoesthesia was found in C6, 7, and 8 dermatomes. Right brachial plexopathy was found from a nerve conduction velocity test, and cervical spine MRI was undertaken. The latter revealed mass lesions at the extraforaminal portion at C5~C6 level, leading to a suggestion of lymphoma infiltration (Figure 2(a)). Under suspicion of neurolymphomatosis, PET-CT was conducted, which showed strong FDG uptake along the right brachial plexus (Figure 2(b)). To exclude thrombus on the subclavian vein, the venous doppler sonography was carried out, which, however, showed no evidence of thrombus. Therefore, we were able to diagnose neurolymphomatosis at the C5~C6 brachial plexus. The patient was treated with radiotherapy with a total dose of 3600 cGy divided into 18 fractions. This relieved the pain, and lesions of previous FDG uptake disappeared on the follow-up PET-CT (Figure 2(c)). Rehabilitation also improved her motor weakness and hypoesthesia. Since her general condition was not good enough to receive further treatment, the patient was placed under supportive care. She died 8 months after her initial diagnosis of NL.

## 3. Discussion

NL is a rare disease, which has not been researched on a large scale, and its incidence has been poorly defined. According to a report by the International Primary CNS Lymphoma Collaborative Group (IPCG), NL developed in 50 patients over a 16-year period. Most cases of NL are related to non-Hodgkin's lymphoma (90%), but some can be found in acute leukemia (10%) [2]. In our study, both cases were DLBCL of NHL; one case was primary NL involving cranial nerves and relapsed into NL of the brachial plexus, and the other was PCNSL and progressed to NL of the brachial plexus. The most common type of lymphoma is DLBCL, followed by follicular lymphoma. NL can occur as a primary disease or as a relapse or progression of a previously treated disease [1].

Despite its low incidence, consideration of neurolymphomatosis is important for diagnosis when a patient complains of related symptoms, especially a patient with a history of hematologic malignancy. Symptoms differ greatly according to the sites involved, and there are also some cases without specific symptoms. According to Baehring et al.'s report, NL presentation has 4 patterns: (1) painful involvement of nerves or roots, (2) cranial neuropathy, with or without pain, (3) painless involvement of peripheral nerves, or (4) painful or painless involvement of a single peripheral nerve [1]. When the brachial plexus is involved, as in the cases described here, arm weakness and hypoesthesia may occur. In the present cases, both patients first experienced pain in the shoulder and upper arm, and hypoesthesia developed later. In a similar case reported by Swarnkar et al., the patient with brachial plexus NL also had shoulder pain and a rapid onset of weakness, numbness, and paresthesia in his arm [4]. According to the IPCG case series, painful neuropathy developed in 76% of NL patients [2]. This suggests that pain may be an important initial symptom of NL in lymphoma patients, especially when it is combined with neurologic symptoms.

In order to diagnose NL, radiological evaluation by, for example, MRI and PET-CT may be effective. The diagnostic sensitivity of MRI is reported to be around 40% [1]. A finding of nerve or root enlargement, with or without contrast enhancement, can be seen on MRI scans [4–6]. However, these signs can be found in other diseases, such as inflammatory radiculopathy, neurofibromatosis, and peripheral nerve sheath malignancy, which makes NL difficult to distinguish from other conditions [2, 7]. In terms of PET-CT, sensitivity for identifying malignant peripheral nerve lesions is 87.5%~100%; it is especially useful in patients who have already been diagnosed with hematologic malignancy [2, 5, 8–10]. PET-CT shows abnormal FDG uptake. Therefore, parallel use of MRI and PET-CT is a more effective means of achieving an early diagnosis of NL. Although biopsy remains the diagnostic gold standard, with a sensitivity of 88%, half of NL patients did not have a biopsy because of difficulties in performing a biopsy of the relevant parts and the possibility of permanent nerve damage and false negative diagnosis [2, 11]. So a biopsy is performed only when diagnosis cannot be made with imaging and CSF examination, and the biopsy alternative is regarded as not being very risky. In the cases discussed in this paper, MRI and PET-CT were carried out as the prime diagnostic method. Cerebrospinal fluid cytological evaluation can be helpful in some patients, although its sensitivity is reported as 21%, which is quite low [1].

The most important clues for diagnosis are a history of non-Hodgkin's lymphoma and abnormal FDG uptake of the lesion. A nerve conduction test is useful for localization of the affected lesion [12].

There is currently no standard treatment for NL. There has been little extensive research on NL, so data comparing effects of regimens are unreliable. Generally, the treatment of NL is similar to that of PCNSL, involving systemic chemotherapy alone or a combination of systemic therapy and intrathecal chemotherapy or radiotherapy [1, 2]. The choice may be made according to the extent of the lymphoma. Our patients were treated with radiotherapy with or without intrathecal chemotherapy.

Rituximab is a monoclonal antibody to the protein CD20 and is used as the first-line regimen of NHL (R-CHOP). However, rituximab is a large molecule, so it cannot permeate the blood-brain barrier or the blood-nerve barrier [13]. In some reported cases, rituximab did not improve the outcome of NL [13]. The optimal regimen for NL has not yet been established, although, in many centers, when an isolated nervous system is involved, high dose of MTX IV with or without a high dose of cytarabine has been administered [1, 2]. When given in high doses, methotrexate can penetrate the blood-brain barrier and blood-nerve barrier and is considered very effective [2]. Although R-CHOP, MCHOD, VAC, and proMACE/cytaBOM are also used for patients with systemic lymphoma, there is no evidence that such a polychemotherapy regimen is more effective than methotrexate monotherapy for the patient who does not have systemic lymphoma [3, 13]. In one of our patients, high dose of MTX was administered after radiotherapy, but the illness continued to progress.

Because lymphoma in our patients was localized in the right brachial plexus, limited-field radiation was an effective means of alleviating symptoms and improving the lesion. However, when the NL infiltrates into the CNS and multiple structures, extensive radiation is less effective and is poorly tolerated in most patients [2].

In a few cases, surgical intervention is needed, such as urgent decompressive laminectomy [14].

There is no standard evaluation of treatment response, but, based on symptom mitigation and improvement of radiographic signs, an improvement of 50~70% was recorded [1, 2]. PET-CT may also be utilized for evaluation of the effect of treatment for NL [15]. In our patients, abnormally strong uptake of FDG along the brachial plexus disappeared, along with symptoms, after concurrent chemotherapy with radiotherapy.

Despite the treatment, the prognosis for patients with NL is poor. The median survival of NL patients-is 10 months after diagnosis [2]. Poor prognosis can arise from the characteristics of the disease, but it can also result from delayed diagnosis or misdiagnosis. In Baehring et al.'s report, autopsies of 145 patients who died from NHL revealed that about 40% of these patients had evidence of peripheral involvement of lymphoma [1]. This suggests that their illness was not correctly diagnosed in their lifetime [12].

 As already mentioned, the absence of a standard regime of management of NL can be one cause of its poor prognosis. Larger studies about NL must be performed, in order to yield the precise incidence, comparison between treatment results, and establishment of the standard management.

Therefore, when a patient who has a history of hematologic malignancy complains of neurologic symptoms, the rare condition of NL should be considered. For early diagnosis and treatment, MRI and PET-CT are very useful. Treatment can be systemic chemotherapy and/or intrathecal chemotherapy with or without radiotherapy. Regardless of treatment, the prognosis for patients with NL is poor.
